# Acceptability of HPV Self-Sampling for Cervical Cancer Screening Among Previously Screened Women in Türkiye: A Cross-Sectional Study

**DOI:** 10.3390/healthcare14101312

**Published:** 2026-05-12

**Authors:** Selda Songur Dağlı, Merve Altun

**Affiliations:** Department of Obstetrics and Gynecology, Kırşehir Ahi Evran University, Kırşehir 40100, Türkiye; merve.altun@ahievran.edu.tr

**Keywords:** HPV, cervical cancer screening, self-sampling, acceptability, screening preferences, public health, Türkiye

## Abstract

**Background:** Cervical cancer remains a major public health problem, particularly in low- and middle-income countries where screening coverage is suboptimal. Human papillomavirus (HPV) self-sampling has emerged as a promising strategy to improve participation in cervical cancer screening programs. This study aimed to evaluate attitudes, preferences, and acceptability regarding HPV self-sampling among Turkish women. **Methods:** This cross-sectional study was conducted at a tertiary care center in Türkiye between January and June 2025. A total of 302 women aged 30–65 years who had previously undergone clinician-collected cervical sampling were included. Participants completed a structured questionnaire assessing their preferences, concerns, and attitudes toward HPV self-sampling. Statistical analyses were performed using SPSS version 29. **Results:** Overall, 73.6% of participants supported the inclusion of HPV self-sampling in the national screening program. However, 49% preferred clinician-collected sampling, while 41.7% preferred self-sampling. Approximately 34.4% reported anxiety related to performing self-sampling. Higher educational level was significantly associated with increased acceptance (*p* = 0.002), whereas age was not significantly associated. **Conclusions:** HPV self-sampling appears to be an acceptable alternative among previously screened women and may help address certain barriers to participation. Further studies are needed to evaluate its real-world implementation and potential role in supporting participation in broader populations.

## 1. Introduction

Cervical cancer remains one of the most preventable malignancies worldwide; however, it continues to represent a significant public health burden, particularly in low- and middle-income countries where screening coverage is suboptimal according to the World Health Organization [1]. Globally, cervical cancer is the fourth most common cancer and the fourth leading cause of cancer-related mortality among women [2]. In response, the WHO has launched a global strategy aiming to eliminate cervical cancer as a public health problem, targeting 90% HPV vaccination coverage, 70% screening coverage, and 90% treatment access [1].

Persistent infection with high-risk human papillomavirus (HPV), particularly types 16 and 18, is the primary etiological factor in cervical carcinogenesis [3]. Consequently, HPV-based screening has become the cornerstone of modern cervical cancer prevention strategies, offering higher sensitivity compared to cytology-based methods [4].

Despite the implementation of organized screening programs, participation rates remain below the desired levels in many countries, including Türkiye [5,6,7]. Barriers to screening are multifactorial and include fear of gynecological examination, embarrassment, cultural factors, lack of awareness, and logistical difficulties [7,8]. Importantly, even in settings where screening is provided free of charge, such as Türkiye, non-structural barriers continue to limit participation [8].

In this context, underserved populations are defined as individuals who face barriers to accessing routine screening despite the availability of services. These include women with limited health literacy, those living in rural or underserved areas, and individuals with sociocultural barriers to gynecological examination [8].

HPV self-sampling has emerged as a promising alternative approach to address these barriers. This method allows women to collect their own vaginal samples in a private setting, potentially increasing participation by improving convenience, autonomy, and acceptability. A growing body of evidence has demonstrated that HPV testing on self-collected samples has comparable diagnostic accuracy to clinician-collected samples when validated assays are used [9,10].

Recent updates in international guidelines increasingly support the integration of self-sampling into cervical cancer screening strategies. The American Cancer Society and consensus recommendations led by Nicolas Wentzensen emphasize the potential role of self-collected HPV testing in improving screening coverage, particularly among under-screened populations [11,12,13,14].

In several countries, such as the Netherlands and Australia, organized self-sampling programs have demonstrated increased participation among women who do not attend routine screening [12,13,14,15]. However, data regarding the acceptability and implementation of HPV self-sampling in Türkiye remain limited.

Therefore, this study aimed to evaluate the acceptability of HPV self-sampling among Turkish women and to identify factors influencing their screening preferences.

## 2. Methods

This cross-sectional study was conducted in 2025 at the gynecology outpatient clinic of Kırşehir Training and Research Hospital, affiliated with Kırşehir Ahi Evran University Faculty of Medicine, Türkiye.

Women aged 30–65 years who had previously undergone clinician-collected cervical sampling were included. A total of 302 participants were enrolled.

Participants were recruited consecutively from eligible women attending the gynecology outpatient clinic during the study period, based on predefined inclusion criteria.

The questionnaire was developed based on previously published studies and consisted of 14 items assessing the participants’ attitudes, preferences, and concerns regarding HPV self-sampling. The draft questionnaire was reviewed by the study investigators for clarity, relevance, and comprehensibility before administration. It was administered in a standardized manner after the participants had received brief verbal information about the HPV self-sampling procedure.

As a questionnaire-based study, the findings may be subject to response bias, including social desirability bias.

Descriptive statistics were used to summarize the data. Associations were analyzed using the Fisher–Freeman–Halton exact test due to small expected cell counts. A *p*-value < 0.05 was considered statistically significant.

This study was designed to evaluate attitudes and acceptability toward HPV self-sampling. No biological samples were collected, and no diagnostic performance outcomes (e.g., sensitivity, specificity, or false-positive rates) were assessed.

## 3. Results

A total of 302 women were included (mean age: 44.76 ± 8.10 years). The sociodemographic and screening-related characteristics of the participants are presented in Table 1.

**Figure 1:**
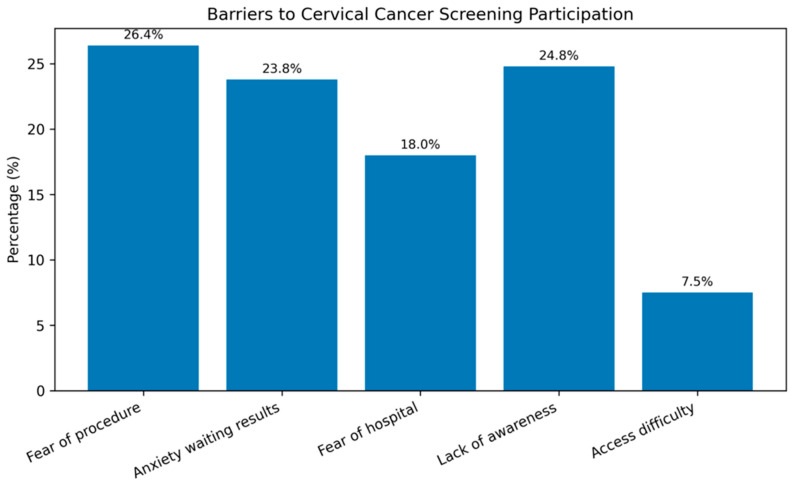
Reasons for not regularly participating in cervical cancer screening.

The most commonly reported barriers to cervical cancer screening were fear of the procedure (26.4%) and limited awareness (24.8%), followed by anxiety while waiting for results (23.8%) and fear of visiting healthcare facilities (18.0%) (Figure 1).

**Figure 2:**
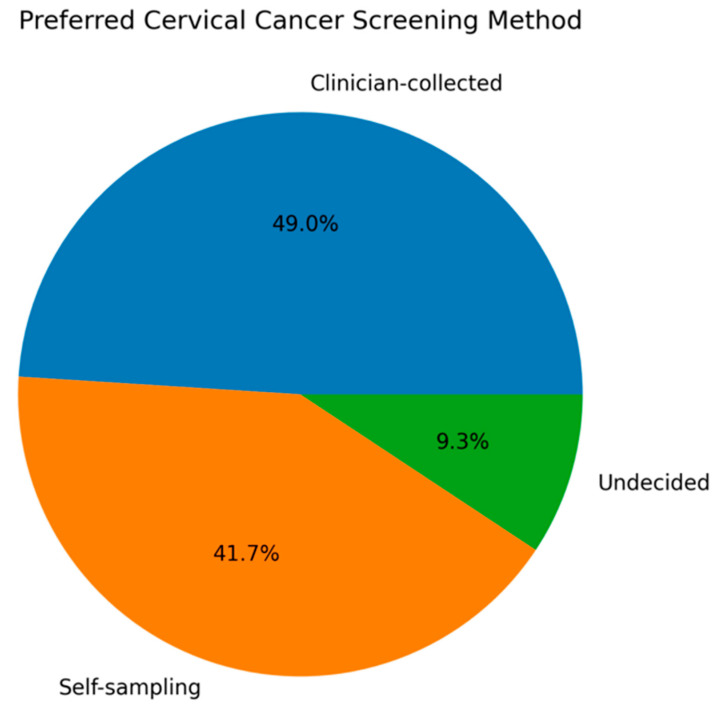
Preferred cervical cancer screening method among participants.

Overall, 73.6% of participants supported the inclusion of HPV self-sampling as a screening option. Additional screening-related characteristics and attitudes toward HPV self-sampling are summarized in Table 2. However, 49% still preferred clinician-collected sampling, while 41.7% preferred self-sampling (Figure 2).

Approximately 34.4% of participants reported that performing self-sampling could cause anxiety.

Educational level was significantly associated with self-sampling preference (*p* = 0.002), with higher acceptance among university graduates. No significant association was found between age and preference (Table 3) Association between educational level and preference for HPV self-sampling.

## 4. Discussion

This study provides evidence that HPV self-sampling is widely accepted among Turkish women and may represent a feasible complementary strategy within cervical cancer screening programs. A substantial proportion of participants (73.6%) supported the inclusion of self-sampling in the national screening program, highlighting its potential role in improving screening accessibility.

The high level of acceptability observed in our study is consistent with previous international studies reporting favorable attitudes toward self-sampling across diverse populations [6,16,17]. These findings suggest that self-sampling is broadly acceptable across different healthcare systems and cultural contexts. Recent systematic review evidence from Asian populations further confirms the high acceptability and feasibility of HPV self-sampling, supporting its applicability across diverse cultural settings [18]. The increased privacy, convenience, and autonomy associated with self-sampling are key factors contributing to its acceptance [6].

However, nearly half of the participants (49%) preferred clinician-collected sampling. This finding is particularly important, as it suggests that self-sampling may be better positioned as a complementary option rather than a replacement for clinician-based screening. Similar patterns have been observed in other studies, where a proportion of women continue to prefer clinician involvement due to perceived reliability and reassurance, despite strong evidence demonstrating the comparable diagnostic accuracy of self-collected samples [10].

The preference for clinician-collected sampling observed in our study may reflect concerns regarding test reliability and confidence in performing the procedure correctly. This finding highlights the importance of trust in healthcare providers and suggests that self-sampling strategies should be accompanied by adequate patient education and reassurance mechanisms [10,16].

Cultural factors and healthcare-seeking behaviors specific to Türkiye may also influence screening preferences. In healthcare systems where physician-led care is traditionally emphasized, individuals may feel more confident in clinician-based procedures, which could explain the continued preference for clinician-collected sampling [7,8].

A notable proportion of participants (34.4%) reported anxiety related to performing self-sampling. This highlights a critical barrier to implementation. Concerns regarding correct sample collection, fear of obtaining inaccurate results, and lack of confidence in performing the procedure have been previously reported [6,16]. These concerns underline the importance of structured patient education, clear instructions, and supportive communication strategies when implementing self-sampling programs.

Educational level was significantly associated with preference for self-sampling, indicating that health literacy plays a key role in the acceptance of alternative screening methods. Women with higher educational levels may have greater confidence in performing self-sampling and interpreting health-related information. This finding is consistent with previous studies demonstrating a positive association between education and acceptance of HPV self-sampling [19].

From a public health perspective, these findings are particularly important. Even in countries such as Türkiye, where cervical cancer screening is provided free of charge, participation remains suboptimal due to non-structural barriers such as fear, embarrassment, and lack of awareness [7,8]. Self-sampling represents a patient-centered approach that may help overcome these barriers, particularly among under-screened populations [6].

The implementation of HPV self-sampling requires robust healthcare system support. International experiences suggest that organized strategies, such as mail-based distribution or community-based approaches, can facilitate participation in cervical cancer screening programs [12,15,20]. However, ensuring appropriate follow-up of positive cases remains a critical challenge. Effective referral systems for colposcopy and treatment are essential to translate improved engagement in screening into better clinical outcomes.

It is important to note that our study population consisted of women who had previously participated in cervical cancer screening. Therefore, the findings may not fully reflect the attitudes of never-screened populations, who are a primary target for self-sampling strategies. Previous studies suggest that the impact of self-sampling may be even greater in these populations [6].

The scarcity of Türkiye-specific data further underscores the importance of the present study, which provides one of the first insights into women’s attitudes toward HPV self-sampling in this setting. These findings may contribute to the development of evidence-based, context-specific screening strategies.

Importantly, these findings suggest that HPV self-sampling should not be considered as a replacement for clinician-based screening but rather as a complementary strategy to address barriers to participation. This approach may be particularly valuable for engaging under-screened populations and supporting the effectiveness of existing screening programs [6,20].

These findings are also aligned with global cervical cancer elimination efforts, which emphasize the importance of increasing screening coverage and reducing barriers to participation through innovative approaches such as self-sampling [1,20].

## 5. Limitations

This study has several limitations. First, it was conducted in a single tertiary care center, which may limit the generalizability of the findings. However, the study population reflects real-world participants within the national screening program, providing valuable insights into practical barriers and attitudes toward HPV self-sampling. Second, all participants had prior experience with clinician-collected sampling, which may influence their preferences. Future multicenter studies including never-screened populations are warranted to further evaluate the impact of self-sampling on screening uptake.

## 6. Conclusions

HPV self-sampling appears to be a well-accepted approach among previously screened women and may help address key barriers related to privacy, accessibility, and discomfort. While clinician-based sampling remains preferred by a substantial proportion of women, self-sampling may serve as a complementary option within cervical cancer screening programs.

From a public health perspective, integrating self-sampling into existing screening strategies may support efforts to improve accessibility and engagement, particularly among under-screened populations. Future multicenter studies are needed to evaluate its real-world implementation and potential impact on screening participation.

## Figures and Tables

**Table 1:** Participant characteristics and screening-related variables (n = 302).

Variable	Value
**Age (years), mean ± SD**	44.76 ± 8.10
**Education level**	
Illiterate	6 (2.0%)
Literate only	6 (2.0%)
Primary school	118 (39.1%)
Secondary school	30 (9.9%)
High school	70 (23.2%)
University	72 (23.8%)
**Occupation**	
Housewife	202 (66.9%)
Health personnel	38 (12.6%)
Other	62 (20.5%)
**Pregnancies, mean ± SD**	2.67 ± 1.0
**Births, mean ± SD**	2.44 ± 0.3
**Abortions, mean ± SD**	0.23 ± 0.5
**Mode of delivery**	
Vaginal delivery	162 (53.6%)
Cesarean delivery	86 (28.5%)
Both vaginal and cesarean	26 (8.6%)
Nulliparous	28 (9.3%)
**Previous cervical cancer screening tests**	
Once	162 (53.6%)
Twice	86 (28.5%)
Three times	26 (8.6%)
Four or more	26 (8.6%)
Do not remember	2 (0.7%)
**Time since last cervical screening test**	
0–2 years	196 (64.9%)
3–4 years	72 (23.8%)
≥4 years	32 (10.6%)
Do not remember	2 (0.7%)
**History of colposcopy**	
Yes	6 (2.0%)
No	288 (95.4%)
Do not remember	8 (2.6%)

**Table 2:** Attitudes toward cervical cancer screening and HPV self-sampling (n = 302).

**Reasons for insufficient participation in screening**	
Fear of procedure	80 (26.4%)
Anxiety waiting for results	72 (23.8%)
Fear of visiting health facility	54 (18.0%)
Limited awareness	75 (24.8%)
Difficulty accessing healthcare	23 (7.5%)
**Place of last cervical cancer screening**	
Cancer screening center (KETEM)	122 (40.4%)
Family physician	72 (23.7%)
Hospital	84 (27.8%)
Private clinic	24 (8.6%)
**Support for self-sampling as an option in the screening program**	
Yes	222 (73.6%)
No	74 (24.5%)
No opinion	6 (2.0%)
**Self-sampling would cause anxiety**	
Yes	104 (34.4%)
No	192 (63.6%)
No opinion	6 (2.0%)
**Preferred screening method if given a choice**	
Clinician-collected sampling	148 (49.0%)
Self-sampling	126 (41.7%)
Undecided	28 (9.3%)

**Table 3:** Association between educational level and preference for HPV self-sampling.

Educational Level	Clinician-Collected Sampling n (%)	Self-Sampling n (%)	Undecided n (%)
Illiterate	4 (2.7)	0 (0.0)	2 (7.1)
Literate only	0 (0.0)	4 (3.2)	2 (7.1)
Primary school	70 (47.3)	34 (27.0)	14 (50.0)
Secondary school	18 (12.2)	10 (7.9)	2 (7.1)
High school	38 (25.7)	28 (22.2)	4 (14.3)
University	18 (12.2)	50 (39.7)	4 (14.3)
Total	148 (49.0)	126 (41.7)	28 (9.3)

*p* = 0.002 (Fisher–Freeman–Halton exact test).

## Data Availability

The data presented in this study are available from the corresponding author upon reasonable request. The data are not publicly available due to ethical and privacy restrictions.
